# Divalproex sodium in severe anaemia: A case report

**DOI:** 10.1186/1745-0179-3-9

**Published:** 2007-07-10

**Authors:** Maneesh Gupta

**Affiliations:** 1 Deaf Mental Health Services, Specialities Directorate, Birmingham and Solihull Mental Health NHS Trust Birmingham, UK

## Abstract

**Background:**

Prescribing psychotropic drugs in clients with severe anaemia has rarely been reported before.

**Case Presentation:**

A 36 year old man with severe anaemia was prescribed divalproex sodium 250 mg daily for mood swings. He had unbearable side effects and had to discontinue the medication in nine days.

**Conclusion:**

Divalproex sodium may result in severe side effects in clients with severe anaemia.

## Background

Prescribing psychotropic medications is a challenge in those whose physiological functions are disturbed. Treating a mental health problem in a patient suffering from severe anaemia, could be even more challenging. There is very little evidence on prescribing in such a clinical scenario and even anecdotal reports and case reports can be used as a guide.

This case report discusses the use of divalproex sodium in a client with severe anaemia.

## Case presentation

Mr AB a 36 year old gentleman, bilateral amputee above knee, with a possible diagnosis of personality disorder had been self harming by cutting himself on the arms for many years. This consistent self harm caused chronic iron deficiency anaemia, which failed to respond to iron supplements. He had consistently refused to take psychotropic medicines because they had either not worked or had given him unbearable side effects. Concerned at his deteriorating haemoglobin levels, we conducted an assessment to ascertain what the client's mental health needs were.

Over 2006, his Hb level had fallen from around 5 g/dl to 4 g/dl (N = 13.5–18.0 g/dl) and he continued to self harm. All other physiological functions (liver, renal) were within normal limits. After some discussion he agreed to start divalproex sodium at 250 mg daily with the aim of gradually increasing the dose, so that his mood swings might be tackled and perhaps help him to reduce self harm. He was not depressed and he did not want any antidepressants. He felt negative towards social services, and mental health services.

After a few days, he began having "hot sweats, scratching (himself), self harm worsening, heart pain in the middle of the chest, twitch movements in arms, loss of appetite, less sleep, worse moods and palpitations". He persisted with the medication for a total of 9 days by when he had also been "scratching (his) head till it was bleeding, having blood in stools, and feeling that (his) skin was turning yellow". In consultation with the mental health team he stopped divalproex sodium. Within three days he reported that his sleep was better, that he did not feel the urge to scratch himself as much as before and that his self harm had returned to the level it was before starting divalproex sodium. He still felt irritable but this remitted in one week, after which he felt things had returned to "normal".

## Discussion

Clients with poor physiological functions pose challenges to clinicians who want to prescribe medications which have not been used in such situations. Severe iron deficiency anaemia is a scenario that is unusual in clinical practice.

This client had a gradual deterioration in his haemoglobin (Hb) level thought to be caused by consistent self harm for many years. He had not responded to antidepressants in the past and had a list of medications to which he had suffered "allergic" reaction to in the past. These included erythromycin, trimipramine, co-amoxiclav, and flupenthixol.

Divalproex sodium was advised to treat his mood swings which had been identified in the past to be of concern. Valproic acid has been used in Diamond-Blackfan anaemia as a therapeutic agent [1,2] although it is known to cause haematological toxicity [3]. No other case reports were found on a Medline/PUBMED search (valproate anaemia; limits: case reports) on the therapeutic use of valproate in a patient suffering from anaemia.

The intensity and the number of side effects that the low dose of divalproex caused in this client, may suggest his sensitivity to the compound. There is mention of patients with valproate associated idiosyncratic drug reactions (IDR) having deficient erythrocyte glutathione peroxidase activity, low plasma selenium concentrations, low COP1 ratios, and low COP2 ratios compared with age-matched controls [4]. We did not measure any of those parameters but it is something that any further anecdotal use could test.

Alternatively, these adverse effects could be a direct or indirect result of his physiological status-severe anaemia. Other clinicians who may be looking at using divalproex sodium in a patient having anaemia should be cautious.

## Conclusion

Divalproex sodium may result in severe side effects in clients with severe anaemia.

## Competing interests

The author has accepted hospitality from various pharmaceutical companies in UK and India. He has accepted honoraria to speak at clinical gatherings from Astra Zeneca and Wyeth Laboratories. The author holds some shares in Indian pharmaceutical companies.
